# National Association of Dental Faculties

**Published:** 1896-10

**Authors:** 


### Reports of Society Meetings.

### NATIONAL ASSOCIATION OF DENTAL FACULTIES.

The Thirteenth Annual Meeting of the National Association of Dental Faculties was held at the Grand Union Hotel, Saratoga Springs, commencing August 1, 1896.

The following colleges were represented:

*Birmingham Dental College.*—T. M. Allen.

*University of Denver, Dental Department.*—W. E. Griswold.

*Columbian University, Dental Department.*—H. C. Thompson.

*National University, Dental Department.*—J. Roland Walton.

*Atlanta Dental College.*—Wm. Crenshaw.

*Southern Medical College, Dental Department.*—Frank Holland.

*Chicago College of Dental Surgery.*—T. W. Brophy and Louis Ottofy.

*Northwestern College of Dental Surgery.*—L. L. Davis.

*Northwestern University Dental School.*—Theo. Menges and Geo. H. Cushing.

*Indiana Dental College.*—G. E. Hunt.

*Louisville College of Dentistry.*—Francis Peabody.

*Baltimore College of Dental Surgery.*—B. Holly Smith.

*University of Maryland, Dental Department.*—F. J. S. Gorgas.

*Boston Dental College.*—J. A. Follett.

*Harvard University, Dental Department.*—Thos. Fillebrown.

*University of Michigan, Dental Department.*—J. Taft.

*Detroit College of Medicine, Dental Department.*—G. S. Shattuck.

*University of Minnesota, College of Dentistry.*—Thos. E. Weeks.

*Kansas City Dental College.*—J. D. Patterson.

*Western Dental College.*—D. J. McMillen.

*Missouri Dental College.*—A. H. Fuller.

*University of Buffalo, Dental Department.*—W. C. Barrett.

*New York College of Dentistry.*—Frank Abbott.

*Cincinnati College of Dental Surgery.*—W. T. McLean.

*Ohio College of Dental Surgery.*—H. A. Smith.

*Cleveland University of Medicine and Surgery, Dental Department.*—S. B. Dewey.

*Western Reserve University, Dental Department.*—H. L. Ambler.

*Pennsylvania College of Dental Surgery.*—C. N. Peirce.

*Philadelphia Dental College.*—T. C. Stellwagen and S. H. Guilford.

*University of Pennsylvania, Dental Department.*—E. C. Kirk.

*University of Tennessee, Dental Department.*—J. P. Gray.

*Vanderbilt University, Dental Department.*—H. W. Morgan and W. H. Morgan.

*University College of Medicine, Dental Department.*—L. M. Cowardin.

*Royal College of Dental Surgeons of Ontario.*—J. B. Willmott.

The following colleges were elected to membership:

*Howard University, Dental Department, Washington, D. C.*—James B. Hodgkin.

*Marion Sims College of Medicine, Dental Department, St. Louis, Mo.*—J. H. Kennerly.

*Dental Department of Tennessee Medical College, Knoxville, Tenn.*—R. N. Kesterson.

The following applications for membership were reported favorably by the Executive Committee for final action next year:

University of Omaha, Dental Department, Omaha, Neb.; Ohio Medical University, Dental Department, Columbus, O.; Baltimore

Medical College, Dental Department, Baltimore, Md.; Dental Department of Milwaukee Medical College, Milwaukee, Wis.

The New York Dental School announced its intention to complete its application next year.

The report of the secretary stated that there were in the United States fifty-three institutions teaching dentistry or conferring the dental degree, as follows: Dental schools in active operation, forty-six; organized during the year, two; in course of organization, one; corporations conferring the dental degree, four. Of the dental colleges, thirty-six were now members of the Association, eight had applications for membership pending, two had signified their intention of applying, and the two newly organized have announced in their catalogues their intention to comply with the rules of the Association.

The report of the Committee on Schools, presented by its chairman, Dr. Follett, stated that reports had been received from thirty-five schools as to their equipment under the resolution adopted last year. These reports showed that the schools were well provided with lecture-rooms, and in most instances with ample laboratory and dispensary accommodations, with sufficient and appropriate appliances. They indicate a broadening in the general course of instruction, as well as fuller courses in all departments. Several colleges have recently added courses in bacteriology and extended their work in histology and pathology in practical ways. During the year 1895–96 the number of matriculates at the thirty-five colleges reporting was five thousand five hundred and thirty-two; graduates, one thousand three hundred and sixty-three.

Mr. Melville Dewey, secretary of the Board of Regents of the University of New York, appeared before the Association by invitation of some of the members, and gave a masterly address on the needs of the movement for higher education in professional ranks. Incidentally, Mr. Dewey explained some of the details of the system pursued in New York, and stated that, greatly to the surprise of those in charge of the various professional educational institutions in the State, the number of students had steadily increased since the higher requirements had been put into force by the Board of Regents.

Among the more important legislation enacted by the Association were the following:

### REGULATING THE ADMISSION OF STUDENTS.

*Preliminary Examination.*

The following preliminary examination shall be required of students seeking Admission to colleges of this Association.

——— ——— HIGH SCHOOL.
——— ——— 189

To the Faculty of ——— ———

M ——— ——— desires to present ———self as a candidate for admission to the Course of Dentistry, ——— ———

He has pursued in this school the branches against which numbers appear,—the numbers being the standings upon a scale of 100. Our course requires five recitations or exercises weekly, in each branch. Our terms are ten weeks in length.

### PRELIMINARY.

2 terms Orthography, standing.
2 terms Reading, standing.
2 terms Writing.
2 terms Arithmetic.
2 terms Geography.
2 terms Grammar.
2 terms History United States.
—
14

These are required in all cases, and fourteen counts given for the same.

### ELECTIVE.

3 terms University Algebra, through Quadratics.

3 terms Geometry, plane and solid.

2 terms Physiology.

2 terms Physical Geography.

1 term Botany, with analysis of forty plants.

3 terms General History.

3 terms Natural Philosophy.

3 terms English Literature.

2 terms Civil Government.

2 terms Rhetoric.

2 terms History of England.

3 terms American Literature.

3 terms Chemistry.

1 term Commercial Arithmetic.

2 terms Astronomy.

2 terms Geology.

2 terms Natural History.

1 term Political Economy.

2 terms Drawing.

3 terms German.

3 terms Greek.

3 terms Latin Reader, Cæsar.

3 terms Cicero, four orations.

3 terms Virgil, six books.

1 term Book-Keeping.

3 terms French.

3 terms Manual Training.

(After session of 1901–1902 United States History becomes elective, and entitles to two credits.)

### FOR THE SESSION OF 1897–98.

| | Counts. |
|---|---|
| Preliminary | 14 |
| Elective | 18 |
| Total | 32 |

FOR THE SESSION OF 1898–99, 1899–1900.

| | Counts. |
|---|---|
| Preliminary | 14 |
| Elective | 27 |
| Total | 41 |

FOR THE SESSION OF 1900–1901.

| | Counts. |
|---|---|
| Preliminary | 14 |
| Elective | 36 |
| Total | 50 |

For the session 1901–1902 and thereafter no preliminary credits; forty-eight credits from the studies classed as elective.

When the text-book mentioned has not been completed, the exact amount of work done should be stated.

The candidate above named is recommended as of good moral character, studious habits, and, judging from past records, able to carry forward the work of a dental college course.

The rules for the admission of students take effect with the session of 1897–98.

—— —— Principal.

ADMISSION TO ADVANCED GRADES ON CERTIFICATES.

The colleges of this association may receive into the advanced grades of juniors and seniors only such students as hold certificates of having passed examinations in the studies of the freshman or junior grades respectively, such certificates to be pledges to any college of the association to whom the holders may apply that the requisite number of terms have been spent in the institutions by which the certificates were issued.

INTERMEDIATE CERTIFICATE.

Place Date

This certifies that ———— has been a member of the ———— class in the ———— during the term of ————

He was examined at the close of the term in the required studies, as stated herein, and is entitled to enter the

| Freshman Year. | Junior Year. |
|---|---|
| [List of Studies.] | [List of Studies.] |

This certificate shall by correspondence be verified by the dean of the college by which it was issued. Without such certificate no student shall be received by any college of this Association for admission to the advanced grade, except on such conditions as would have been imposed by the original school, and these to be ascertained by conference with the school whence he came.

### LIMITING THE TIME FOR THE RECEPTION OF STUDENTS.

No member of this Association shall give credit for a full course to students admitted later than ten days after the opening day of the session, as published in the announcement.

In case one is prevented by sickness, properly certified by a reputable practising physician, from complying with the foregoing rule, the time of admission shall not be later than twenty days from the opening day.

In cases where a regularly matriculated student, on account of illness, financial condition, or other sufficient cause, abandons his studies for a time, he may re-enter his college at the same or subsequent session, or where under similar circumstances he may desire to enter another college, then with the consent of both deans he may be transferred, but in neither case shall he receive credit for a full year unless he has attended not less than seventy-five per cent. of a six months' course of lectures.

### ATTENDANCE, EXAMINATIONS.

Attendance upon three full courses of not less than six months each in separate academic years shall be required before examination for graduation. The year shall be understood to commence August 1, and end the following July 31.

Beginning with the session of 1896–97, the examinations conducted by the colleges of this Association shall be in the English language only.

A student who is suspended or expelled for cause from any college of this Association shall not be received by any other college during that current session. In case the action of the first college is expulsion, the student shall not be given credit at any time for the course from which he was expelled. Any college suspending any student shall at once notify all other members of this Association of its action.

### APPLICATIONS FOR MEMBERSHIP.

Applications for membership in this Association shall be made in writing, favorably indorsed by the faculties of two or more colleges of the Association and the Board of Dental Examiners of the State in which it is located.

Such application shall then be referred to a special committee of three which shall be appointed by the chair upon each application. The duty of this committee shall be to visit the school applying during its session, personally examine its facilities for teaching, methods of instruction, and efficiency of the faculty, and report to the Executive Committee, which report shall, if favorable, be acted upon.

Each application shall be accompanied by a sum of money sufficient to defray the expenses of the special committee.

The constitution was so amended that hereafter it will require a two-thirds vote instead of a majority to elect new members.

The following resolution, offered by Dr. Peirce, was on motion adopted:

Whereas, In view of various reports frequently being circulated derogatory to the character of certain schools without any one being willing to prefer charges sustaining such statements.

*Resolved*, That the Executive Committee be and is hereby authorized to exercise full power to investigate all such innuendoes or charges by visiting the school or schools, or authorize some one to perform this duty; summoning witnesses, etc., in order that all such statements shall be sustained or proved false.

*Resolved*, That a sum to be determined by the officers, president, secretary, and treasurer, be and is hereby appropriated for the purpose of paying expenses essential to the carrying out of the provisions of the above resolution.

The following communication from the National Association of Dental Examiners was read and on motion adopted:

*Resolved*, That this Association requests the National Association of Dental Faculties to enact a rule prohibiting colleges from receiving beneficiary students recommended by State boards and associations.

The following, offered by Dr. Abbott, was adopted:

*Resolved*, That the committee of three appointed by the chair to report on applications for membership shall determine and report to this Association at its next meeting the minimum requirements of such colleges as desire to become members of this Association as to length of course, plant, equipment, facilities for teaching, and the number and efficiency of its faculty.

Dr. Brophy offered the following, which was adopted:

*Resolved*, That a graduate of a recognized dental college, who applies to a college of this Association for the degree of Doctor of Dental Surgery or Doctor of Dental Medicine, shall complete one full course of instruction in said college and comply with all other requirements of the senior class.

The following lie over till next year for final action:

Offered by Dr. Barrett:

*Resolved*, That after the regular session of 1897–98 the annual college term for the members of this Association shall be seven full months.

*Resolved*, That it is advisable that the National Association of Dental Faculties in future meet in connection with the National School of Dental Technics at a time of year when the colleges are in session, and before the time for the issuance of the annual catalogues.

A committee, consisting of Drs. Patterson, H. W. Morgan, and Kirk, appointed to consider the advisability of adopting the academic cap and gown for commencement day, reported in favor of adopting the intercollegiate system and in favor of lilac as the distinguishing color for dental schools. Laid over till next year.

The following were elected officers for the ensuing year: J. P. Gray, Nashville, Tenn., President; Truman W. Brophy, Chicago, Vice-President; Louis Ottofy, Chicago, Secretary; Henry W. Morgan, Nashville, Tenn., Treasurer.

*Executive Committee.*—J. Taft, Cincinnati; Thomas Fillebrown, Boston; B. Holly Smith, Baltimore, Md.

*Ad Interim Committee.*—H. A. Smith, Cincinnati; Thomas E. Weeks, Minneapolis; J. D. Patterson, Kansas City, Mo.

The newly-elected officers were installed, and the president announced the standing committees as follows:

*Committee on Text-Books.*—S. H. Guilford, Philadelphia, Pa.; J. B. Willmott, Toronto, Canada; Theodore Menges, Chicago, Ill.; L. M. Cowardin, Richmond, Va.; James Truman, Philadelphia, Pa.

*Committee on Schools.*—J. A. Follett, Boston, Mass.; G. E. Hunt, Indianapolis, Ind.; C. N. Peirce, Philadelphia, Pa.; A. H. Fuller, St. Louis, Mo.; D. J. McMillen, Kansas City, Mo.

Adjourned.

